# CysC as a predictor of left ventricular remodeling and major adverse cardiovascular events in patients with acute myocardial infarction

**DOI:** 10.3389/fcvm.2025.1698109

**Published:** 2025-12-12

**Authors:** Ni An, Yanmei Yuan, Hailong Lu, Tian Liu, Defeng Pan

**Affiliations:** 1 Department of Geriatrics, The Affiliated Hospital of Xuzhou Medical University, Xuzhou, China; 2 Department of Cardiology, The Affiliated Hospital of Xuzhou Medical University, Xuzhou, China

**Keywords:** acute myocardial infarction, Cystatin C, left ventricular remodeling, major adverse cardiovascular events, percutaneous coronary intervention

## Abstract

**Background:**

Percutaneous coronary intervention (PCI) is the primary revascularization method for acute myocardial infarction (AMI); however, patients remain at significant risk of developing left ventricular remodeling (LVR), which is closely associated with major adverse cardiovascular events (MACE). This study evaluated cystatin C (CysC) as a potential biomarker for LVR and MACE after PCI in AMI patients.

**Methods:**

A total of 168 AMI patients who underwent PCI were followed for 6 months. Transthoracic echocardiography was performed at admission and 6-month follow-up. The endpoints were LVR and MACE. Multivariable logistic regression analysis was used to identify factors associated with LVR, while Cox proportional hazards regression was employed for time-to-MACE analysis. Variables with *P* < 0.1 in univariate analyses were included in multivariate models. Stepwise forward selection was applied to construct the final models, with adjustment for potential confounders.

**Results:**

22% of patients developed LVR and 18.6% experienced MACE. Multivariate regression analysis identified CysC as independently associated with both LVR and MACE after PCI. The AUC for CysC in predicting LVR and MACE was 0.757 and 0.707, respectively. Adding CysC to conventional risk prediction models improved their discriminatory accuracy. Internal validation using bootstrap sampling (1,000 replications) confirmed model reliability. Kaplan–Meier analysis stratified by CysC tertiles demonstrated a significant association between increasing CysC levels and higher incidence of MACE (log-rank *P* = 0.002).

**Conclusion:**

CysC was independently associated with LVR and MACE after PCI in patients with AMI, highlighting its potential value as a biomarker for early identification of high-risk patients and guiding targeted therapeutic interventions.

## 1 Introduction

Acute myocardial infarction (AMI) is a leading cause of death worldwide. Effective treatment strategies aim to limit infarct size, prevent adverse left ventricular remodeling (LVR), and reduce subsequent heart failure development (1). Primary percutaneous coronary intervention (PCI) represents the most effective approach for rapid myocardial reperfusion, although the procedure can sometimes induce reperfusion injury (2). Post-AMI prognosis is largely determined by the extent of permanent myocardial damage and the development of LVR, which occurs in approximately 30% of ST-elevation myocardial infarction (STEMI) patients treated with primary PCI (3). Following AMI, reduced myocardial blood flow leads to tissue necrosis, creating a mechanically weak area that requires scar formation to prevent cardiac rupture. While this initial adaptive remodeling is essential for short-term survival, progressive pathological ventricular dilation and dysfunction often develop at both infarct and remote sites, ultimately contributing to poor clinical outcomes (4). Consequently, identifying patients at high risk for post-PCI LVR is crucial for effective risk stratification and targeted therapeutic interventions in AMI management.

Several biomarkers have demonstrated associations with LVR and major adverse cardiovascular events (MACE). Inflammatory markers such as high-sensitivity C-reactive protein (hs-CRP) and soluble Suppression of Tumorigenicity 2 (sST2) have shown predictive value (5, 6), as have traditional myocardial injury markers including creatine kinase isoenzyme (CK-MB) and N-terminal pro-B-type natriuretic peptide (NT-proBNP) (7). Cystatin C (CysC), a biomarker less influenced by age, gender, and muscle mass compared to conventional renal markers (8), has garnered significant interest as a potential predictor across various disease states (9). Elevated CysC levels correlate with inflammation, oxidative stress, endothelial dysfunction, and atherosclerotic plaque severity, suggesting a critical role in cardiovascular and metabolic disease progression (10). Experimental evidence demonstrates that under stress conditions, CysC can penetrate cardiomyocytes and influence cardiac remodeling through modulation of pro-hypertrophic signaling via the MAPK pathway (11). Despite these insights, the utility of CysC for risk stratification and prediction of LVR development in AMI patients following PCI remains inadequately explored and warrants further investigation.

## 2 Materials and methods

### 2.1 Study population

**Figure 1:**
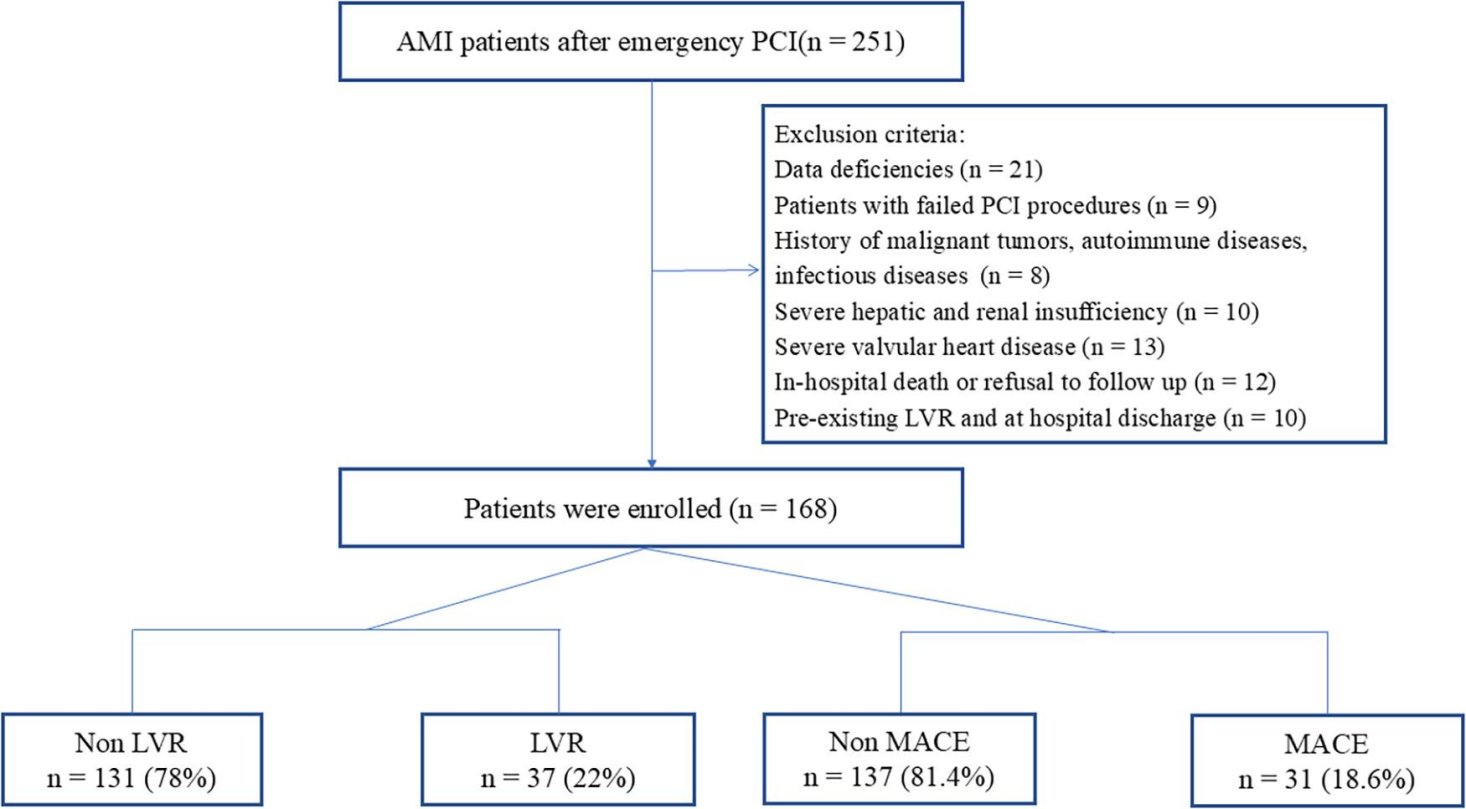
Study flowchart.

This retrospective study included consecutive AMI patients who received emergency PCI at the Affiliated Hospital of Xuzhou Medical University between January 2023 and June 2024. Inclusion criteria: (1) age 18–80 years; (2) meeting the diagnostic criteria for AMI (12); (3) successful PCI completion within 24 h of symptom onset; and (4) availability of echocardiographic data at baseline (within 1 week of admission) and at 6-month follow-up. Exclusion criteria included: in-hospital death or refusal to participate in follow-up; coexisting malignant tumors, autoimmune diseases, or active infections; severe structural heart diseases; severe hepatic or renal failure; pre-existing left ventricular remodeling; and incomplete clinical data. This retrospective study was approved by the Ethics Committee of the Affiliated Hospital of Xuzhou Medical University (Figure 1).

### 2.2 Study follow-up

Patients were followed for 6 months post-PCI through telephone interviews and outpatient clinic visits. This study had two clinical endpoints: (1) Left ventricular remodeling (LVR) was defined as an increase in echocardiographic left ventricular end-diastolic volume (LVEDV) of more than 20% between baseline (within 1 week after admission) and 6-month follow-up (7). Patients were categorized into LVR (*n* = 37) and non-LVR (*n* = 131) groups based on this criterion. (2) Major adverse cardiovascular events (MACE) included composite events of all-cause death, reinfarction, mechanical complications of infarction, severe cardiac failure, and major hemorrhage.

Patients were followed for 6 months post-PCI through telephone interviews and outpatient clinic visits. This study had two clinical endpoints: (1) LVR was defined as an increase in echocardiographic left ventricular end-diastolic volume (LVEDV) of more than 20% between baseline (within 1 week after admission) and 6-month follow-up (7). Patients were categorized into LVR (*n* = 37) and non-LVR (*n* = 131) groups based on this criterion. (2) MACE included composite events of all-cause death, reinfarction, mechanical complications of infarction, severe cardiac failure and ischemic stroke. All MACE events were adjudicated by two independent cardiologists through comprehensive review of medical records, discharge summaries, and follow-up data. Any discrepancies between the adjudicators were resolved through consensus discussion. Patients were divided into MACE (*n* = 31) and non-MACE (*n* = 137) groups based on follow-up outcomes.

### 2.3 Data collection

(1) Baseline information: age, gender, smoking history, body mass index (BMI), systolic blood pressure (SBP), diastolic blood pressure (DBP), medication history, hypertension, diabetes mellitus, Killip classification, ST-segment elevation. (2) Angiographic and surgical characteristics: culprit vessels (IRA-LCX, IRA-LAD, IRA-RCX) and preoperative Thrombolysis in Myocardial Infarction (TIMI) risk score. (3) Laboratory parameters: cystatin C (CysC), platelet (PLT), white blood cell (WBC), hemoglobin (HGB), albumin (ALB), high sensitivity c-reactive protein (hs-CRP), glucose (Glu), hemoglobin A1c (HbA1c), N-terminal pro B-type natriuretic peptide (NT-proBNP), high-sensitivity troponin T (hs-TnT), creatine kinase isoenzyme (CK-MB), lipid indicator, alkaline phosphatase (ALP), creatinine (Scr) and uric acid (UA).

### 2.4 Transthoracic echocardiography

Collect and sort the results of ultrasonic heart dynamic diagrams within one week and six months of discharge from the PCI procedure. The patient lies on his or her left side, breathes normally, and measures the left ventricular long shaft cut surface at the sternum's border. The patients' left ventricular remodeling indices were obtained: left atrial diameter (LAD), left ventricular end-diastolic inner diameter (LVEDD), right ventricular diameter (RVD), interventricular septal thickness (IVST), left ventricular posterior wall thickness at end diastole (LVPWTd), and left ventricular ejection fraction (LVEF).

The patient's left ventricular mass index (LVMI) was calculated according to Devereux's formula. Body surface area (BSA)(male) = 0.0057 × height (m) + 0.0121 × weight (kg) + 0.0882, BSA (female)= 0.0073 × height (m) + 0.0127 × weight (kg) - 0.2106. LVM (g) = 1.04 × [(IVST + LVPWTd + LVEDD)3 - LVEDD3] - 13.6; LVMI (g/m2) = LVM/BSA.

### 2.5 Statistical methods

Data were analyzed using SPSS 27.0, R Studio, and Graphpad Prism. Normality was determined using the Kolmogorov–Smirnov test. Normally distributed measures were expressed as mean ± standard deviation (x ± s), and an independent *t*-test was used to compare two groups. Non-normally distributed measures were expressed as median (quartile) [M (P25, P75)], and the Mann–Whitney *U*-test was used to compare two groups. Count data were reported as a percentage, and the *χ*^2^ test was performed to compare two groups. The link between LVR and CysC was investigated using Spearman rank correlation analysis. The independent risk factors for the occurrence of LVR and MACE were investigated using univariate and multivariate regression analysis, and the odds ratio (OR) and 95% confidence interval (CI) were obtained. Significant indicators from univariate analysis (*P* < 0.1) were included in multivariate regression analysis. The predictive value of CysC for LVR and MACE, as well as its cut-off value, were determined using receiver operating characteristic (ROC) curves. Kaplan–Meier survival curves were produced, and survival rates were compared using the log-rank test. *P* < 0.05 was considered a statistically significant difference. To further explore the dose-response relationship between CysC and clinical outcomes (LVR and MACE), restricted cubic spline (RCS) regression analysis with three knots was performed.

Missing data management: Patients with incomplete clinical information or those lost to follow-up were excluded from the analysis. Given our relatively small sample size, we did not employ imputation methods to avoid introducing bias. For regression analyses, we assessed multicollinearity using variance inflation factors (VIF), with VIF <5 considered acceptable. For Cox regression models, we verified the proportional hazards assumption using Schoenfeld residuals tests and log-log survival plots. Model fit was evaluated using the Hosmer-Lemeshow test for logistic regression and by examining residuals for Cox models. Bootstrap sampling with 1,000 replications was performed to validate model stability and prevent overfitting, as shown in Figure 5.

## 3 Results

### 3.1 Comparison of general information and laboratory indicators

The study included 168 patients, with in the 37 (22%) LVR group and 131 (78%) in the non-LRA group. The difference in NT-proBNP, hs-CRP, Scr, CysC, ALP, left ventricular remodeling indexes (LAD, IVST, LVEDD, LVPWTd, LVEF, LVMI), and MACE was statistically significant (*P* < 0.05). However, the difference in other baseline data was not statistically significant (*P* > 0.05) (Table 1).

**Table 1:** Baseline data comparison between groups.

Variables	Non LVR (*n* = 131)	LVR (*n* = 37)	*P*
Clinical characteristics			
Age, (years)	57.20 ± 13.05	57.92 ± 11.87	0.763
Male, *n* (%)	111 (84.73)	29 (78.38)	0.360
BMI, (kg/m^2^)	25.73 ± 3.46	26.68 ± 3.36	0.139
Systolic blood pressure, (mm/Hg)	124.00 (113.00, 137.00)	129.00 (111.00, 148.00)	0.346
Diastolic blood pressure, (mm/Hg)	79.00 (70.00, 84.00)	78.00 (71.00, 86.00)	0.954
Heart rate, (times/min)	78.00 (70.00, 86.00)	80.00 (72.00, 94.00)	0.166
Laboratory characteristics			
Peak CK-MB, (ng/L)	143.20 (49.65, 296.55)	132.60 (82.60, 300.00)	0.775
Peak hs-TNT, (ng/L)	2,535.00 (1131.00, 5146.50)	2,804.00 (1797.00, 10000.00)	0.160
Peak NT-proBNP, (pg/mL)	1,213.00 (580.85, 2246.50)	2,098.00 (744.00, 3096.00)	0.044*
Peak hs-CRP, (mg/L)	27.20 (10.50, 54.55)	47.80 (24.40, 77.80)	0.012*
TC, (mmol/L)	4.18 ± 1.10	4.37 ± 0.94	0.357
TG, (mmol/L)	1.55 (1.04, 2.08)	1.34 (1.04, 1.63)	0.185
sdLDL-C, (mmol/L)	0.80 (0.55, 1.12)	0.76 (0.58, 1.06)	0.829
Lp(a), (mg/L)	237.00 (115.50, 348.00)	256.00 (132.00, 533.00)	0.223
HbA1c, (%)	5.90 (5.50, 6.60)	5.90 (5.60, 6.70)	0.568
Glucose, (mg/dL)	5.97 (5.10, 6.85)	5.85 (5.51, 7.67)	0.169
WBC, (10^9/L)	10.34 ± 2.73	10.77 ± 2.95	0.408
PLT, (10^9/L)	216.11 ± 56.55	230.85 ± 63.04	0.174
HGB, (g/L)	136.00 (126.50, 147.00)	140.00 (128.00, 149.00)	0.550
Scr, (umol/L)	61.00 (51.00, 67.00)	70.00 (51.00, 83.00)	0.038*
ALB, (umol/L)	37.70 (35.95, 40.35)	38.50 (36.50, 41.20)	0.419
UA, (U/L)	311.00 (246.50, 359.50)	351.00 (276.00, 408.00)	0.092
eGFR, (mL/min/1.73 m^2^)	117.89 (104.05, 120.00)	107.89 (89.32, 120.00)	0.101
CysC (mg/L)	−0.19 (−0.79, 0.20)	0.52 (0.11, 0.82)	<.001*
ALP, (U/L)	76.00 (64.50, 85.00)	68.00 (61.00, 78.00)	0.046*
Cardiovascular risk factors			
Smoking, *n* (%)	67 (51.15)	18 (48.65)	0.789
Hypertension, *n* (%)	57 (43.51)	20 (54.05)	0.256
Diabetes, *n* (%)	33 (25.19)	12 (32.43)	0.380
Echocardiographic indicators			
LAD, (mm)	36.00 (34.00, 39.00)	39.00 (36.00, 42.00)	0.003*
IVST, (mm)	9.00 (8.00, 9.50)	10.00 (9.00, 10.00)	0.002*
LVEDD, (mm)	49.00 (47.00, 52.00)	52.00 (49.00, 56.00)	0.007*
LVPWTd, (mm)	9.00 (8.00, 9.00)	10.00 (9.00, 10.00)	<.001*
RVD, (mm)	22.00 (21.00, 23.00)	22.00 (21.00, 23.00)	0.757
LVEF, (%)	56.00 (50.50, 61.00)	51.00 (48.00, 56.50)	0.006*
LVMI	85.68 (75.67, 96.60)	93.13 (86.63, 108.10)	<.001*
Clinical presentation			
Killip class ≥2, *n* (%)	19 (14.50)	5 (13.51)	0.879
ST-segment elevation	92 (70.23)	29.(78.38)	0.329
Days of hospitalization	6.00 (5.00, 7.00)	6.00 (5.00, 7.00)	0.598
Angiographic characteristics			
IRA-LCX, *n* (%)	39 (29.77)	8 (21.62)	0.329
IRA-LAD, *n* (%)	51 (38.93)	19 (51.35)	0.176
IRA-RCA, *n* (%)	40 (30.53)	10 (27.03)	0.680
Pre-TIMI ≤ 1, *n* (%)	103 (78.63)	27 (72.97)	0.468
Clinical drug			
Statins, *n* (%)	129 (98.47)	35 (94.59)	0.211
Sacubitril Sodium Tablets, *n* (%)	54 (41.22)	21 (56.76)	0.093
ACEI/ARB, *n* (%)	31 (23.66)	7 (18.92)	0.542
*β*-blockers, *n* (%)	116 (88.55)	32 (86.49)	0.956
Spironolactone, *n* (%)	14 (10.69)	8 (21.62)	0.143
Antiplatelet drug, *n* (%)	130 (99.24)	37 (100.00)	1.000
Outcome			
MACE	17 (12.98)	14 (37.84)	<.001*

“*” Significant correlation at the 0.05 level (two-tailed); BMI, body mass index; TC, serum total cholesterol; TG, serum triglyceride; sdLDL, small and dense low-density lipoprotein; Lp(a), Lipoprotein(a); NT-proBNP, N-terminal pro B-type natriuretic peptide; hs-TnT, high-sensitivity troponin T; Peak CK-MB, peak creatine kinase-MB; peak hs-CRP, peak high sensitivity c-reactive protein; Scr, serum creatinine; eGFR, estimated glomerular filtration rate; WBC, white blood cell; HGB, hemogloin; PLT, platelet; ALB, albumin; UA, uric acid; ALP, alkaline phosphatase; CysC, cystatin C; LCX, left circumflex branch; LAD, left anterior descending branch; RCA, right coronary artery; LAD, left atrial diameter; LVEDD, left ventricular end-diastolic inner diameter; RVD, right ventricular diameter; IVST, interventricular septal thickness; LVPWTd, left ventricular posterior wall thickness at end diastole; LVEF, left ventricular ejection fraction; LVMI, left ventricular mass index; TIMI, thrombolysis in myocardial infarction; ARB, angiotensin II receptor antagonist; ACEI, angiotensin-converting enzyme inhibitors; MACE, major adverse cardiovascular events.

### 3.2 Correlation between CysC and predictive indicators of LVR

Spearman correlation analysis revealed that CysC showed significant positive correlations with hs-TnT, NT-proBNP, Scr, IVST, LVPWTd, and LVMI, and a significant negative correlation with eGFR (*P* < 0.05) (Table 2).

**Table 2:** Correlation between cysC and predictive indicators of LVR.

Parameter	Correlation coefficient (r)	*p*-value
Peak hs-TNT, (ng/L)	0.170*	0.028
Peak NT-proBNP, (pg/mL)	0.354**	<.001
Peak hs-CRP, (mg/L)	0.126	0.104
Scr, (umol/L)	0.376**	<.001
UA, (U/L)	0.225**	0.003
eGFR, (mL/min/1.73 m2)	−0.467**	<.001
LAD, (mm)	0.084	0.279
IVST, (mm)	0.171*	0.027
LVEDD, (mm)	0.063	0.420
LVPWTd, (mm)	0.164*	0.034
RVD, (mm)	−0.098	0.207
LVEF, (%)	−0.134	0.082
LVMI	0.201**	0.009

“*” Significant correlation at the 0.05 level (two-tailed); “**” Significant correlation at the 0.01 level (two-tailed); hs-TnT, high-sensitivity troponin T; NT-proBNP, N-terminal pro B-type natriuretic peptide; peak hs-CRP, peak high sensitivity c-reactive protein; Scr, serum creatinine; UA, uric acid; eGFR, estimated glomerular filtration rate; LAD, left atrial diameter; LVEDD, left ventricular end-diastolic inner diameter; RVD, right ventricular diameter; IVST, interventricular septal thickness; LVPWTd, left ventricular posterior wall thickness at end diastole; LVEF, left ventricular ejection fraction; LVMI, left ventricular mass index.

### 3.3 Clinical end points - LVR

#### 3.3.1 Univariate and multivariate logistic regression analysis of LVR in patients with AMI

Univariate logistic regression analysis was performed with LVR as the dependent variable and NT-proBNP, hs-CRP, Scr, CysC, ALP, LVEF, and LVMI as independent variables. Variables with *P* < 0.1 in univariate analysis were included in the multivariate model. Stepwise regression (forward LR) identified CysC and LVMI as independent predictors of LVR (OR > 1, *P* < 0.05) (Table 3).

**Table 3:** Logistic regression analysis of LRA.

Variables	Univariate Logistic Regression Analysis		Multivariate Logistic Regression Analysis	
	*P*	OR (95%CI)	*P*	OR (95%CI)
Peak hs-CRP	0.066	1.01 (1.00∼1.02)		
Peak NT-proBNP	0.016	1.01 (1.01∼1.01)		
Scr	0.037	1.02 (1.01∼1.05)		
ALP	0.110	0.98 (0.96∼1.00)		
CysC	<.001	2.48 (1.60∼3.85)	0.002	2.34 (1.35∼4.04)
LVEF	0.018	0.94 (0.90∼0.99)		
LVMI	<.001	1.05 (1.02∼1.08)	0.034	1.04 (1.01∼1.07)

NT-proBNP, N-terminal pro B-type natriuretic peptide; Peak-hsCRP, peak high sensitivity c-reactive protein; Scr, serum creatinine; ALP, alkaline phosphatase; CysC, cystatin C; LVEF, left ventricular ejection fraction; LVMI, left ventricular mass index.

#### 3.3.2 Predictive value of LVR in patients with AMI

**Figure 2:**
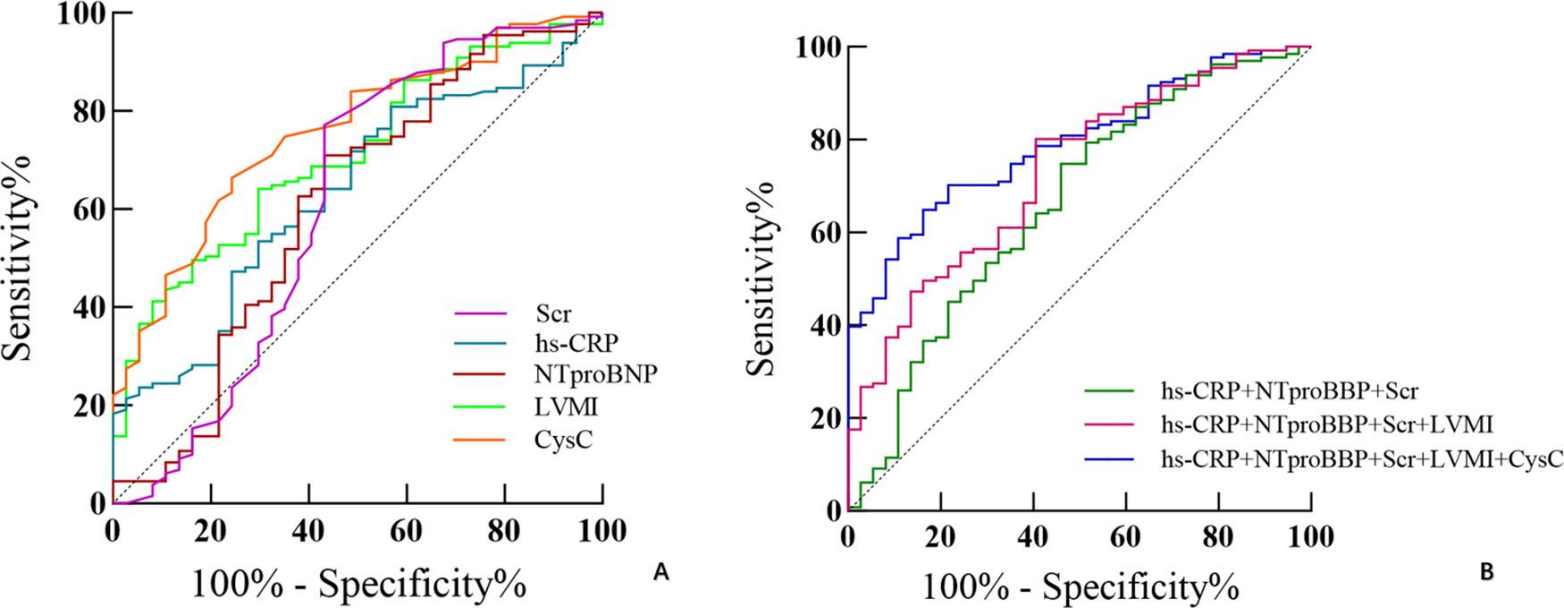
ROC of combined parameters for identifying LVR. **(A)** Predictive performance of single parameters. **(B)** Predictive performance of parameter combinations. The combination of CysC with the baseline model improved the predictive value.

ROC curve analysis demonstrated that CysC predicted LVR with an AUC of 0.757 (sensitivity 75.7%, specificity 66.4%). The AUCs for LVMI, hs-CRP, NT-proBNP, and Scr were 0.709, 0.635, 0.609, and 0.612, respectively. The combined model (CysC + LVMI + hs-CR*P* + Scr + NT-proBNP) yielded an improved AUC of 0.792 (sensitivity 83.8%, specificity 64.9%) (Table 4; Figure 2).

**Table 4:** ROC analysis of LVR prediction.

Parameter	Cutoff	AUC	95% CI	*P*	Sensitivity (%)	Specificity (%)
CysC	0.885	0.757	0.675∼0.839	<0.001	75.7	66.4
LVMI	89.834	0.709	0.624∼0.797	<0.001	70.3	64.1
hs-CRP	65.6	0.635	0.539∼0.732	0.012	43.2	80.9
Scr	67.5	0.612	0.489∼0.735	0.038	56.8	77.1
NT-proBNP	1,898.26	0.609	0.495∼0.723	0.043	56.8	71.0
hs-CRP + Scr + NT-proBNP	—	0.663	0.559∼0.768	0.002	51.4	74.8
LVMI + hs-CRP + Scr + NT-proBNP	—	0.730	0.642∼0.819	<0.001	59.5	80.2
CysC + LVMI + hs-CRP + Scr + NT-proBNP	—	0.792	0.721∼0.863	<0.001	83.8	64.9

NT-proBNP, N-terminal pro B-type natriuretic peptide; Peak-hsCRP, peak high sensitivity c-reactive protein; Scr, serum creatinine; CysC, cystatin C; LVMI, left ventricular mass index.

### 3.4 Clinical end points - MACE

#### 3.4.1 Univariate and multivariate Cox regression analysis of MACE in patients with AMI

There were 137 patients in the MACE group (81.5%) and 31 in the non-MACE group (18.5%). Cox regression analysis with age, NT-proBNP, CysC, LVEF, and LVMI as independent variables identified CysC and LVMI as significant risk factors for MACE (*P* < 0.05) (Table 5).

**Table 5:** Cox regression analysis of MACE.

Variables	Univariate Cox Regression Analysis		Multivariate Cox Regression Analysis	
	*P*	HR (95%CI)	*P*	HR (95%CI)
Age	0.048	0.01 (0.01∼0.01)		
Peak NT-proBNP	0.006	0.01 (0.01∼0.01)		
CysC	<.001	0.12 (0.06∼0.17)	0.008	0.08 (0.02∼0.15)
LVEF	0.044	−0.01 (−0.02∼−0.01)		
LVMI	<.001	0.01 (0.01∼0.01)	0.029	0.01 (0.01∼0.01)

NT-proBNP, N-terminal pro B-type natriuretic peptide; LVEF, left ventricular ejection fraction; CysC, cystatin C; LVMI, left ventricular mass index.

#### 3.4.2 Predictive value of MACE in patients with AMI

**Figure 3:**
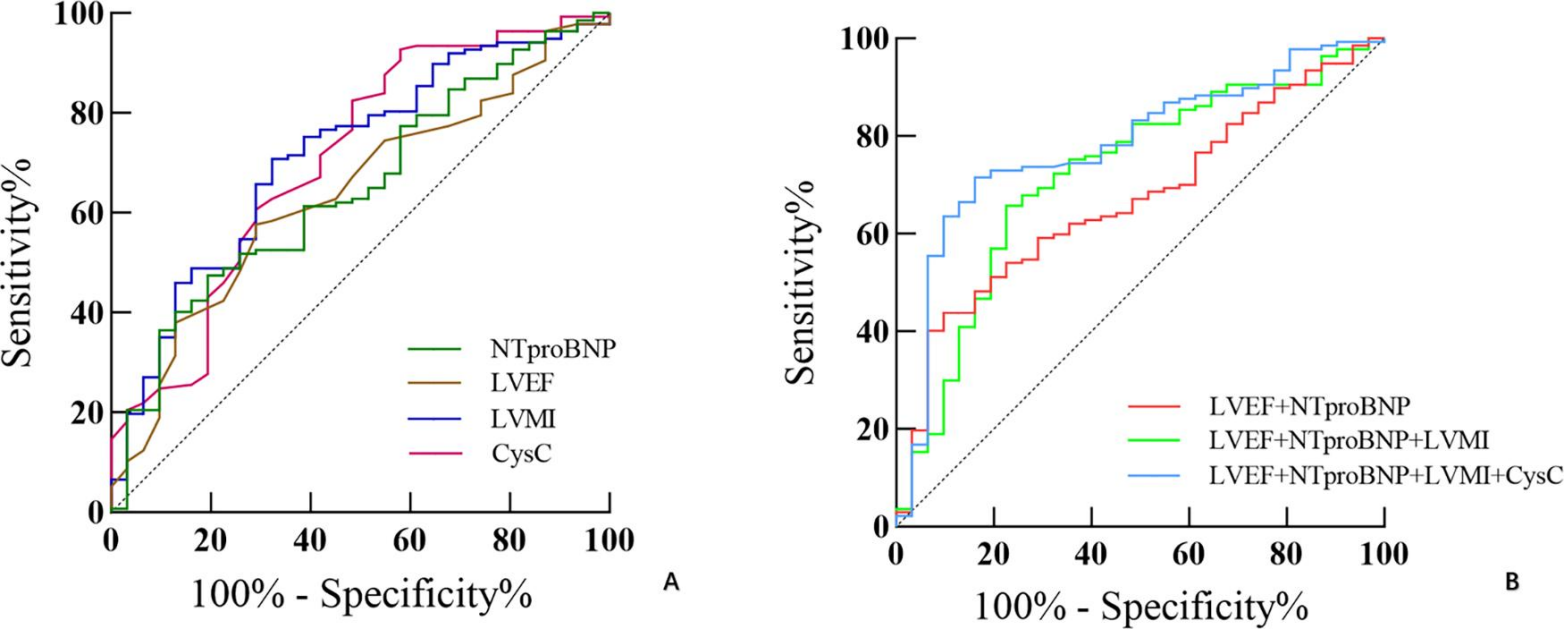
ROC of combined parameters for identifying MACE. **(A)** Predictive performance of single parameters. **(B)** Predictive performance of parameter combinations. The combination of CysC with the baseline model improved the predictive value.

ROC curve analysis identified CysC, LVMI, LVEF, and NT-proBNP as significant predictors of MACE, with respective AUCs of 0.707, 0.714, 0.629, and 0.648 (*P* < 0.05). The combined model incorporating these variables demonstrated superior predictive performance compared to individual markers (Table 6; Figure 3).

**Table 6:** ROC analysis of MACE prediction.

Parameter	Cutoff	AUC	95%CI	*P*	Sensitivity (%)	Specificity (%)
CysC	1.005	0.707	0.600∼0.813	<0.001	41.9	92.7
LVMI	92.706	0.714	0.616∼0.812	<0.001	67.7	70.8
LVEF	54.5	0.629	0.528∼0.730	0.025	29.0	42.3
NT-proBNP	1,073	0.648	0.564∼0.750	0.001	80.6	47.4
LVEF + NT-proBNP	—	0.663	0.559∼0.768	0.002	51.4	74.8
LVEF + NT-proBNP + LVMI	—	0.722	0.621∼0.822	<0.001	77.4	65.7
LVEF + NT-proBNP + LVMI + CysC	—	0.781	0.694∼0.868	<0.001	83.9	71.5

NT-proBNP, N-terminal pro B-type natriuretic peptide; LVEF, left ventricular ejection fraction; CysC, cystatin C; LVMI, left ventricular mass index.

#### 3.4.3 Kaplan–Meier survival curve analysis

**Figure 4:**
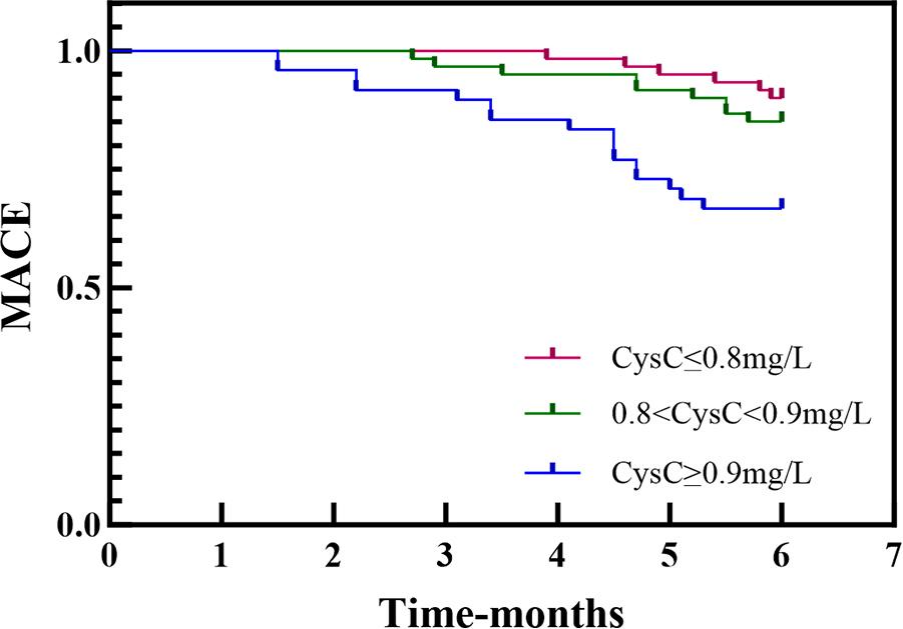
Kaplan-Meyer analysis-Kaplan-Meier curves showing the risk of MACE stratified by CysC. Patients were stratified into three groups based on admission CysC levels (≤0.8 mg/L, 0.8-0.9 mg/L, and ≥0.9 mg/L). The results demonstrate that higher CysC levels were significantly associated with an increased incidence of MACE.

CysC value tertiles were used to classify AMI patients into the CysC ≥0.9 group (*n* = 48), the 0.8 < CysC < 0.9 group (*n* = 60), and the CysC ≤ 0.8 group (*n* = 60). Kaplan–Meier analysis revealed a significant association between increasing CysC levels and higher cumulative incidence of MACE (log-rank *χ*^2^ = 12.21, *P* = 0.002) (Figure 4).

### 3.5 Bootstrap sampling internal validation

**Figure 5:**
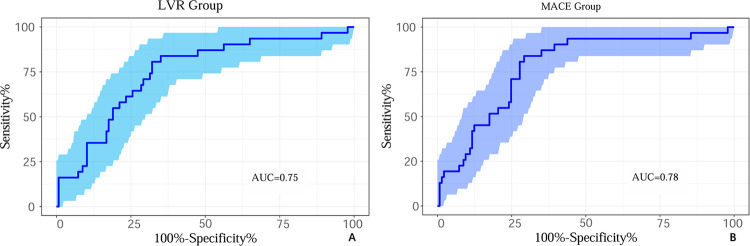
1,000 bootstrap ROC curves. LVR: Bootstraped uncached ROC Curve with 31 positive and 137 negative samples. The AUC is 0.75. MACE: Bootstraped uncached ROC Curve with 31 positive and 137 negative samples. The AUC is 0.78.

Due to the limited sample size, bootstrap sampling with 1,000 replications was performed to validate model reliability and prevent overfitting. For LVR prediction, the combined model (CysC + LVMI + hs-CRP + Scr + NT-proBNP) showed a bootstrap-validated AUC of 0.75. For MACE prediction, the combined model (CysC + LVMI + LVEF + NT-proBNP) yielded a bootstrap-validated AUC of 0.78. These values closely approximated the original model performance, confirming robustness and reliability (Figure 5).

### 3.6 Restricted cubic spline analysis

**Figure 6:**
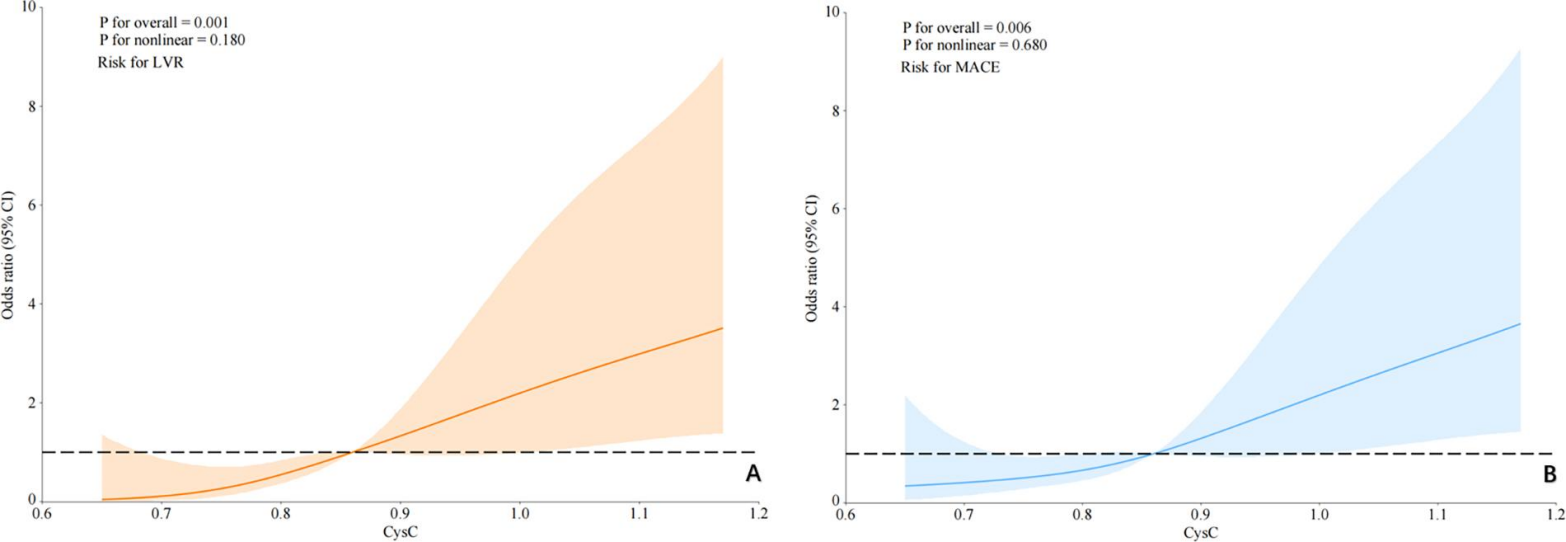
RCS for the odds ratio of the risk. Restricted cubic spline analyses show the association of serum CysC concentrations with the risk of **(A)** LVR and **(B)** MACE. The solid lines represent the odds ratios, and the shaded areas represent the 95% confidence intervals.

RCS analysis was performed to characterize the dose-response relationship between CysC and clinical outcomes. CysC demonstrated a nonlinear association with LVR risk (*P* for nonlinear = 0.180) and a linear association with MACE risk (*P* for nonlinear = 0.680) (Figure 6).

## 4 Discussion

Our study demonstrates that elevated CysC levels are independently associated with LVR and MACE after PCI in AMI patients, highlighting its potential value as an early biomarker. CysC emerges as a critical mediator in the pathogenesis of adverse cardiac remodeling following AMI through multiple interconnected biological pathways. LVR represents a pathological alteration of cardiac structure following AMI that significantly impacts patient prognosis. Despite successful reperfusion therapy with PCI, approximately 20% of patients develop progressive left ventricular dilation that evolves from compensated to decompensated states, ultimately leading to heart failure and increased mortality (13, 14). Current evidence indicates that LVEF reduction remains strongly associated with adverse remodeling and poor outcomes after AMI (15), highlighting the critical need for early biomarkers that can identify high-risk patients before irreversible structural changes occur.

The biological mechanisms underlying CysC's association with adverse remodeling involve complex interactions with cardiac tissue. CysC promotes pathological extracellular matrix remodeling through disruption of the MMP/TIMP balance, particularly by inhibiting cathepsins that regulate collagen degradation, ultimately leading to abnormal fibrosis coexisting with areas of inadequate repair and ventricular wall thinning, which collectively contribute to pathological ventricular dilation (16). Our observation of significant positive correlations between CysC and structural parameters (IVST r = 0.171, *p* = 0.027; LVPWTd r = 0.164, *p* = 0.034; LVMI r = 0.201, *p* = 0.009) provides clinical evidence supporting this mechanistic link. Furthermore, CysC exacerbates post-infarct inflammation by promoting macrophage infiltration and stimulating pro-inflammatory cytokine production (IL-6, TNF-α), creating a microenvironment conducive to progressive remodeling (17). This inflammatory pathway is reinforced by our finding that CysC significantly correlates with hs-TNT (r = 0.170, *p* = 0.028) and NT-proBNP (r = 0.354, *p* < 0.001), established markers of myocardial injury and wall stress (18, 19). Additionally, experimental studies have shown that under stress conditions, CysC can penetrate cardiomyocytes and influence cardiac hypertrophy by activating the MAPK signaling pathway (11), directly linking CysC to cellular mechanisms of ventricular remodeling.

While previous literature has reported seemingly contradictory roles of CysC in cardiovascular disease (20, 21) our data consistently demonstrate its risk-factor function in the acute phase of AMI. This context-dependent behavior may be explained by CysC's dual role in different pathophysiological stages: protective in chronic atherosclerosis through anti-protease activity but detrimental in acute injury through pro-fibrotic and pro-inflammatory actions. Our RCS analysis further reveals a nonlinear dose-response relationship between CysC and adverse outcomes (Figure 6), suggesting threshold effects in its pathological influence that warrant further investigation (22).

CysC's prognostic value extends beyond its role as a simple renal function marker (23–25). The significant improvement in discriminatory accuracy when combining CysC with LVMI (AUC increased from 0.757 to 0.792 for LVR) reflects their complementary pathophysiological roles—CysC representing biochemical processes of remodeling while LVMI captures structural consequences. This finding aligns with previous studies by Ix et al. (26) and Patel et al. (27), who demonstrated associations between elevated CysC and LV hypertrophy independent of renal function, lending credence to CysC's direct involvement in cardiac structural changes.

Our regression analysis revealed that blood CysC and LVMI at admission were independently associated with LVR and MACE following PCI in AMI patients. Changes in left ventricular mass have been correlated with clinical outcomes (28, 29). The significant correlations between CysC and multiple structural and functional cardiac parameters demonstrate its central role in the remodeling process. Although CysC's individual discriminatory value for MACE prediction is somewhat lower than LVMI, combining CysC with conventional markers (hs-CRP, Scr, NT-proBNP) substantially improved risk stratification. Internal validation using bootstrap sampling confirmed the stability and reliability of our prediction model. Kaplan–Meier analysis demonstrated a dose-dependent relationship between CysC levels and MACE incidence, with significantly higher event rates in the highest tertile.

Our investigation found that CysC was strongly correlated with hs-TnT, NT-proBNP, Scr, IVST, LVPWTd, LVMI, and eGFR. Among these parameters, IVST, LVPWTd, and LVMI reflect changes in ventricular morphology; whereas hs-TnT, NT-proBNP, Scr and eGFR have been widely established as predictors of cardiovascular events and mortality in AMI patients (18, 19, 30, 31). These correlations collectively highlight CysC's integrative role in linking cardiac structural remodeling, renal dysfunction, and myocardial injury pathways, providing a comprehensive biological explanation for its strong association with LVR and MACE observed in our study.

These findings have important clinical implications. Measuring CysC at admission could help identify AMI patients at high risk for adverse remodeling before echocardiographic changes become apparent, potentially guiding more aggressive therapeutic interventions. The biological pathways linking CysC to remodeling also suggest potential therapeutic targets, such as MMP inhibitors or anti-inflammatory approaches, though these require further investigation (32, 33). In conclusion, CysC serves as a robust biomarker that reflects active biological processes driving LVR post-AMI. Its integration into clinical risk models could enable earlier identification of high-risk patients and pave the way for novel mechanism-based therapies.

Our research also has some limitations. First and foremost, this is a single-center, retrospective study. There may be consequences from selection bias and mixed factors, limiting the universality and trustworthiness of the results. Most available study samples are restricted to specific patient populations (AMI patients), and people with more significant cardiovascular disease require additional verification. Second, the study relied on a single CysC measurement at admission and provided no evidence of dynamic tracking of its effects on LVR. Future research should focus on CysC's dynamic changes and interactions with other biomarkers in order to improve the accuracy of prognostic assessments of patients with AMI and establish the best strategies for its use in various clinical circumstances. In addition, the expression of CysC varies significantly between biological samples (for example, serum vs. urine), and its mechanism is not entirely understood (34). Finally, the small sample size and short follow-up time necessitate large-scale, multicenter prospective studies to validate CysC's generalizability and prognostic value in different AMI patient groups, to obtain more accurate risk stratification, and to investigate optimization models of treatment strategies.

## 5 Conclusion

CysC was independently associated with LVR and MACE after PCI in patients with AMI, highlighting its potential value as a biomarker for early identification of high-risk patients and optimization of therapeutic strategies.

## Data Availability

The raw data supporting the conclusions of this article will be made available by the authors, without undue reservation.
